# The sound of silence: Binding and retrieval of silence in distractor-response binding

**DOI:** 10.3758/s13414-026-03261-4

**Published:** 2026-05-05

**Authors:** Maria Nemeth, Christian Frings, Philip Schmalbrock, Birte Moeller

**Affiliations:** 1 Department of Psychology, Trier University, Trier, Germany; 2 Institute for Cognitive Affective Neuroscience (ICAN), Trier University, Trier, 54286 Germany

**Keywords:** Action control, Event files, Perception, Absence, Event representation, Silence

## Abstract

In human action control, executing a response is assumed to integrate features of the response and the stimulus into short-term episodic traces known as event files. Repeating any of the features comprised in the event file in a later episode leads to retrieval of other integrated features and can influence current behavior. Event files can include task-relevant features, and task-irrelevant features also can be bound to responses, termed distractor–response binding. Distractor–response binding effects have been shown in multimodal settings, such as for auditory distractors and visual targets. Recently, it has been suggested that silence can be perceived rather than just being cognitively inferred as the absence of sound. In the present study (combined *N* = 120), we used auditory distractors in a distractor–response binding task and investigated whether silence as a distractor (i.e., the absence of presented sound) can be bound to a response and subsequently retrieve this response from an event file. We found that silence as a distractor produced a typical distractor–response binding effect, which, furthermore, did not differ in size from the distractor–response binding effect when two sounds were used as distractors. The present findings indicate that silence operates similarly to sound as an auditory distractor in binding and retrieval in action control and support the notion that moments of absence can elicit perceptual event representations.

Every day, humans navigate through a dynamically changing environment shaped by the continuous influence of different modalities. Whereas irrelevant or aversive visual objects can be easily ignored by simply looking away, irrelevant sounds are harder to avoid, as we cannot simply “hear away.” Given this characteristic of the auditory system, the control of irrelevant auditory information may be equally or even more important than managing irrelevant visual information (Moeller et al., 2012). Indeed, accumulating evidence indicates that not only currently relevant but also task-irrelevant information becomes associated with actions and bias subsequent behavior when a similar episode is later encountered again (e.g., Frings et al., 2007, 2013).

Within the action-control literature, such influences of the immediate past on current actions can be explained by the binding and retrieval in action control framework (BRAC; Frings et al., 2020). Specifically, it is assumed that whenever we plan and execute a response, stimuli features (e.g., shape, color, location) and response features (e.g., response direction, effector) occurring in close temporal proximity are automatically integrated into a common episodic representation of an action episode, a so-called event file (Hommel, 1998, 2004; see Frings et al., 2024, for consensus definitions). An event file is an internal representation of bindings between stimuli and responses (responses and effects), which can be decoded from patterns in neuronal activity (Beste et al., 2023; Eggert et al., 2022). Because the event file is maintained for several seconds (depending on the type of binding; see Hommel & Frings, 2020), it can be retrieved from memory in later episodes upon repetition of any of its components (e.g., a stimulus feature), thereby influencing current actions (Hommel, 1998, 2004).

The binding of task-irrelevant distractor stimuli is commonly investigated in the distractor–response binding paradigm (Frings et al., 2007). Here, dependencies between two consecutive action episodes are investigated within a prime–probe sequence. In a prime, participants perform a choice reaction-time task where they respond to target stimuli (e.g., letters) by pressing either a left or right key, while the targets are accompanied by distractors (e.g., flanker letters from a different stimulus set than targets). The co–occurrence of the distractor and the response in the same prime event is assumed to result in their binding and integration into an event file. Following this integration process, distractor–response bindings can affect subsequent responding in probe depending on the compatibility of the retrieved (prime) and the current (probe) action episode. Specifically, a repetition of the prime distractor in probe is assumed to trigger retrieval of the bound response. When the response also repeats, the retrieved response matches the currently required response, resulting in improved performance (e.g., faster reaction times and/or lower error rates in case of full feature repetition). These performance benefits are assumed to result from the automatic response retrieval from memory, thereby eliminating the need for computational processes of a currently required response (Henson et al., 2014; Logan, 1988). On the other hand, when only the distractor is repeated but the response has to be changed, retrieval of the bound response leads to a mismatch with the currently required response, resulting in impaired performance (e.g., slower response times and/or higher error rates), known as partial repetition costs (Hommel, 1998). Accordingly, the distractor–response binding characteristic of performance facilitation versus impairment can only be observed by jointly considering response relations and distractor relations from prime to probe. Statistically, the effect therefore emerges as an interaction between factors response relation (repetition vs. change) and distractor relation (repetition vs. change).

The distractor–response binding is a robust phenomenon and has been demonstrated for visual (e.g., Frings et al., 2007), tactile (e.g., Moeller & Frings, 2011), and auditory distractors (e.g., Moeller et al., 2012; for a review see Frings et al., 2014). Importantly, evidence suggests that repeating a distractor in a modality different from the target modality (e.g., an irrelevant auditory stimulus and a visual target) is capable of initiating a retrieval process (see also Frings et al., 2014). Although binding effects have most consistently been observed in reaction times, they have also been reported in error rates (e.g., Frings et al., 2007; Moeller & Frings, 2021a). Importantly, both measures are considered independent and equally valid indicators of binding and retrieval processes. Which measure is more sensitive appears to depend on task demands and participants’ speed–accuracy settings (Frings et al., 2020; Selimi & Moeller, 2024).

While the binding literature has traditionally focused on the integration of physically present and directly perceivable features, a growing body of research in perception and action control addresses the question of how the cognitive system represents the *absence of events*. In the literature on auditory perception, this question has been discussed under the label “perception of silence”. One theoretical position holds that silence is not itself perceived but merely cognitively inferred as the absence of sound (O’Shaughnessy, 2008; see also Marr, 2010). In contrast, others have argued that silence can be considered as a perceptual auditory “feature” (i.e., we can hear silence, e.g., Skrzypulec, 2022; Sorensen, 2008). Recent psychophysical evidence supports the latter view. Using temporal illusion paradigms, Goh et al. (2023) showed that silent intervals embedded in auditory contexts elicited temporal distortions perfectly analogous to the illusions produced by sounds. The authors concluded that auditory event representations can emerge even in the absence of positive acoustic content, suggesting that silence can be viewed as a perceptual feature.

Converging evidence comes from the action control literature, supporting the assumption of differentiated and feature-based event representations of absence that can affect cognitive processes in action control. In prevention action tasks, participants act to prevent a specific sensory effect from occurring—for instance, to maintain silence by preventing auditory events from occurring (e.g., Pfister et al., 2021). Intriguingly, it was shown that silence can indeed have features of the event that are not being perceived, in that preventing a long tone leads to significantly longer prevention actions than preventing a short tone (Tonn et al., 2023, Experiment 2). Related work on nonaction-effect binding further demonstrated that intentionally omitted responses can become associated with their contingent effects, much like overt actions (Kühn & Brass, 2010a, 2010b; Weller et al., 2017). These findings align with theories of negation in memory, according to which representations of absent or negated events preserve the identity of what is negated (e.g., “not-left” rather than “nothing”) and are accompanied by a negation tag (e.g., Gilbert, 1991; Wegner et al., 1985).

Together, research on silence perception and action control converges on the view that the cognitive system can form feature-specific representations of absent events. Crucially, the assumption of silence operating at a perceptual level, rather than relying on abstract, conceptually inferred codes, is particularly interesting in the light of the binding and retrieval framework. While event files can incorporate abstract and semantic information (e.g., Frings et al., 2013; Moeller et al., 2016; Singh et al., 2019), evidence suggests that conceptual features are bound and retrieved only when they are attended and task-relevant, whereas mere repetition of unattended conceptual distractors seems to be insufficient to trigger retrieval (Laub & Frings, 2020b; Laub et al., 2021; Singh et al., 2018). Thus, according to the current state of literature, whether silence can be bound to and retrieve responses from event files should critically depend on whether silence is processed as a perceptual feature. If this is the case, it would not only be relevant to the ongoing debate about silence as a perceptual feature but also would provide new insights into action control processes (i.e., whether silence operates as a physical feature in binding and retrieval).

## The present study

The present study investigated whether silence, when occurring as a task-irrelevant auditory distractor, can be integrated with and retrieve responses in the same way as sound. We conducted two experiments, in which we used an adapted version of the distractor–response binding task (Frings et al., 2007). Against the theoretical background of silence perception (Goh et al., 2023), we used visual targets that were accompanied by auditory distractors.

**Fig. 1:**
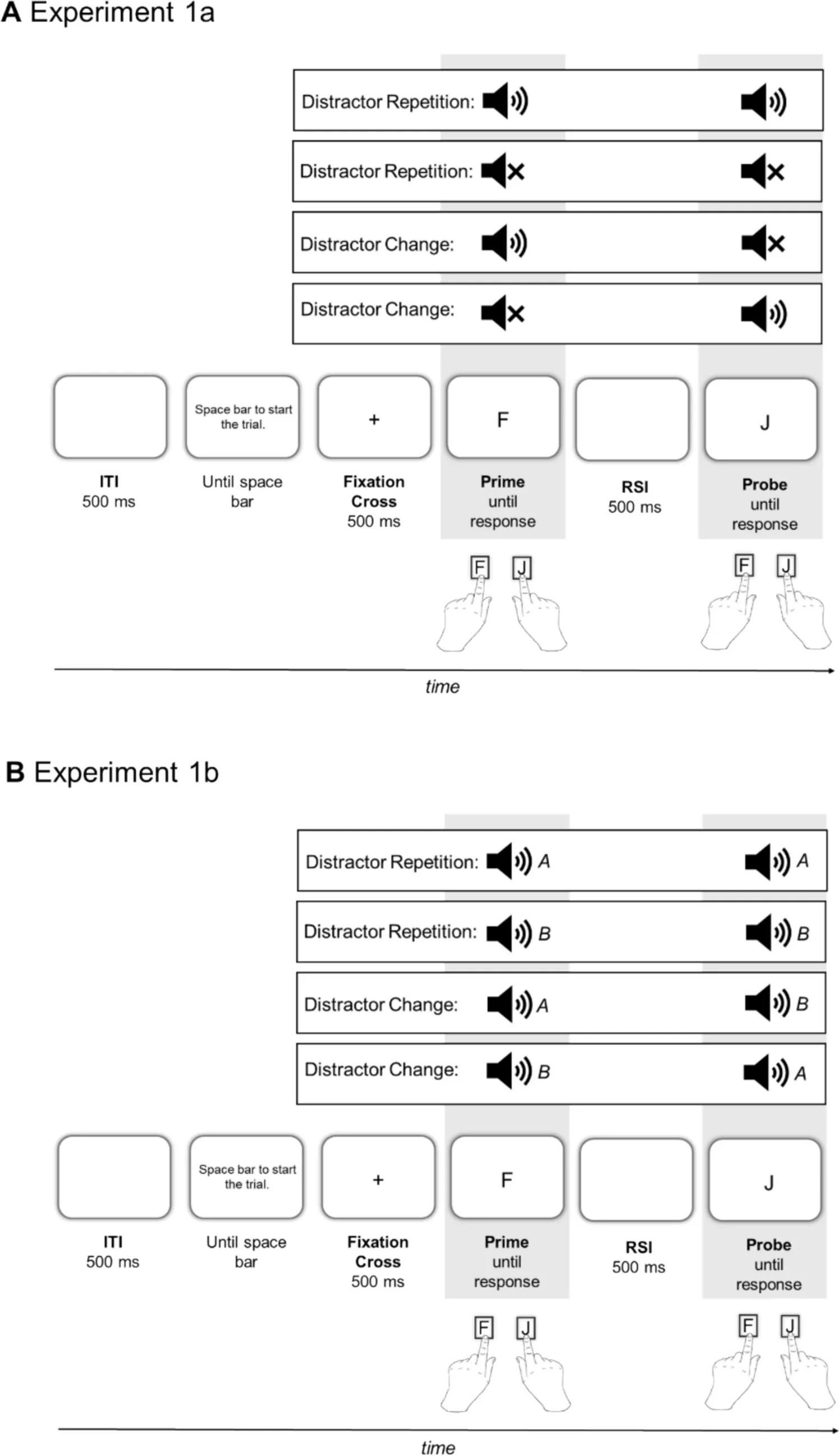
Sequence of events in one trial. Participants responded with their left and right index fingers to the identity of individually presented letters. Target letters were F or J. **A** In Experiment 1a, distractors were silence (i.e., no presented sound) or a sine wave tone. **B** In Experiment 1b, distractors were two different sine wave tones. In response repetition trials (RR), the prime target was repeated as the probe target. In response change trials (RC), the probe target was different from the prime target. In distractor repetition trials (DR), the prime distractor was repeated as the probe distractor. In distractor change trials (DC), the probe distractor was different from the prime distractor. Depicted are response change trials along with all possible distractor repetition and distractor change trials for both experiments. Stimuli are not drawn to scale. RSI = response–stimulus interval. ITI = intertrial interval

Measuring silence as a feature in distractor–response binding is somewhat challenging, however. In a 2 × 2 design, with distractor relation and response relation as within-subjects factors, it is not possible to separately calculate binding effects for sound versus silence. To compute a *pure* distractor–response binding effect for silence, at least two different silence conditions would be required—for instance, for the distractor change conditions (see Fig. 1). Because silence seems to be special in that it is a unique condition, we needed an alternative approach. One way to compare distractor–response binding effects for sound versus silence is to compare distractor–response binding effects that are based on two different sounds versus distractor–response binding effects that are based on one sound and silence. If distractor–response binding effects are observed in an experiment with one sound and silence, then silence is treated as just another sound.

Thus, in Experiment 1a, we investigated whether a typical distractor–response binding effect occurs when using silence and a sine wave tone as distractors. That is, a distractor repetition could either be silence in prime and probe or sound in prime and probe, distractor changes could either be silence in prime and sound in probe or vice versa. In Experiment 1b, we measured distractor–response binding effects when two sine wave tones were used as sound distractors. That is, a distractor repetition could either be sound A in prime and probe or sound B in prime and probe, distractor changes could either be sound A in prime and sound B in probe or vice versa.

We hypothesized that if silence can be bound as an auditory distractor and retrieve integrated responses later on, a typical distractor–response binding effect should emerge in Experiment 1a, even though no actual sound was presented as one of the distractors. Furthermore, if response integration and retrieval differed for silence and sound distractors, we would have to expect a significant difference between binding effects in Experiments 1a and 1b.

## Experiment 1a

### Method

#### Participants

The sample size was calculated according to previous studies investigating distractor–response binding effects, which typically led to medium-sized effects (*d*_z_ ~ 0.50; e.g., Frings et al., 2007; Moeller & Frings, 2014; Schmalbrock et al., 2021). To find an effect with a power of 1 − β = .95 (assuming α = .05, two-tailed; Faul et al., 2007), we needed at least *N* = 54 participants. Accordingly, we recruited 60 participants of Trier University (55 women, three men, two not disclosed, 58 right-handed) with a median age of 21 years (range: 18–31 years) to account for possible dropouts. Participants were recruited via Trier University’s participant platform (Sona Systems; sona-systems.com). All participants consented via online form before participating and received partial course credit as compensation.

#### Design

The design comprised two within-subject factors: response relation (response repetition vs. response change) and distractor relation (distractor repetition vs. distractor change).

#### Apparatus and stimuli

The experiment was programmed using PsychoPy (Peirce et al., 2019; Version 2023.2.3) and ran online via Pavlovia (Peirce & MacAskill, 2018). Instructions and stimuli were shown in white (font: Arial; font size 35 pixels) on a grey background (RGB_255_: 0, 0, 0). Two displays were presented in each trial: a prime and a probe display (see Fig. 1). In both displays, a single target letter was presented at the screen center. Target letters were either a *J* or an *F*. Distractors could either be no presented sound (i.e., silence) or a sine wave tone (250 Hz), which was created using Audacity (Audacity, 2014).

#### Procedure

Instructions were presented at the center of the screen. Participants were instructed to place their index fingers of both hands on the keys *F* and* J*. Their task was always to press the key corresponding to each individually presented letter: using their left index finger for the letter *F* and their right index finger for the letter *J*. It was emphasized that responses were to be made as fast as possible while maintaining high accuracy.

At the beginning of the experiment, a sound test consisting of four trials was conducted. In each trial, a display with the instruction “Listen...“ (original in German, “Hören...”) was shown for 5 s, accompanied either by a sine wave tone or by no sound (i.e., silence). After each display, participants were either asked whether they heard a sound or if silence was presented. They had to respond to the questions by using the *F* key for “yes” and the *J* key for “no.” The questions about sound or silence were varied orthogonally with the presented condition. If participants did not answer all four questions correctly, the sound test was repeated until they answered all four questions correctly.

Next, a training consisting of 16 trials was completed before the main experiment. For participants who made more than 15% errors, the training block was repeated. In training trials, participants received performance-contingent feedback after prime and probe training displays (for a correct response: “correct”; for a wrong response: “WRONG!”; Original in German, “richtig” and “FALSCH!”). During the main experiment, participants only received error feedback immediately following the erroneous response.

The experimental block consisted of 384 trials. All visual stimuli were presented at the center of the screen. Each trial started with the instruction to start the trial by pressing the space bar, followed by the presentation of a fixation mark ( +) at the center (see Fig. 1). The presentation of the prime display followed—that is, the target letter accompanied continuously by the auditory distractor (no sound; i.e., silence, or a sine wave tone), until response detection. The prime response was followed by a 500-ms blank space (response-stimulus interval; RSI), and after that, the probe display appeared. The structure of the probe display was identical to the prime display. Between the trials, a blank screen appeared for 500 ms (intertrial interval; ITI), before the next trial could be started.

The two factors response relation and distractor relation were varied orthogonally. In response repetition trials, the same response required in the prime was also required in the probe. In response change trials, a different response was required in prime and probe. In distractor repetition trials, the prime distractor was again presented in the probe. In distractor change trials, the prime distractor was different from the probe distractor.

### Results

Data processing and analysis were done with R (Version 4.3.0; R Core Team, 2019). The distractor–response binding effect was computed as the distractor repetition benefit in response repetition trials minus the distractor repetition benefit in response change trials ([Response: Repetition / Distractor: Change − Response: Repetition / Distractor: Repetition] − [Response: Change / Distractor: Change − Response: Change / Distractor: Repetition]). For the analysis of reaction times (RTs), trials with incorrect responses in prime or probe were excluded (13.4% of the trials). Additionally, we excluded 2.5% trials with probe RTs below 200 ms and > 1.5 interquartile ranges above the third quartile of each participant’s RT distribution (Tukey, 1977). Thus, 15.9% of trials were excluded from analyses. See Table 1 for mean RTs and error rates.

**Table 1:** Mean response times (in ms) and mean error rates (in percentages) in Experiment 1a and Experiment 1b

	Experiment 1a		Experiment 1b	
Response relation	Distractor repetition	Distractor change	Distractor repetition	Distractor change
Response repetition Response change	392 (0.4) 480 (13.4)	403 (0.5) 483 (10.4)	389 (1.2) 475 (17.5)	406 (1.4) 481 (12.7)

#### Reaction times

**Fig. 2:**
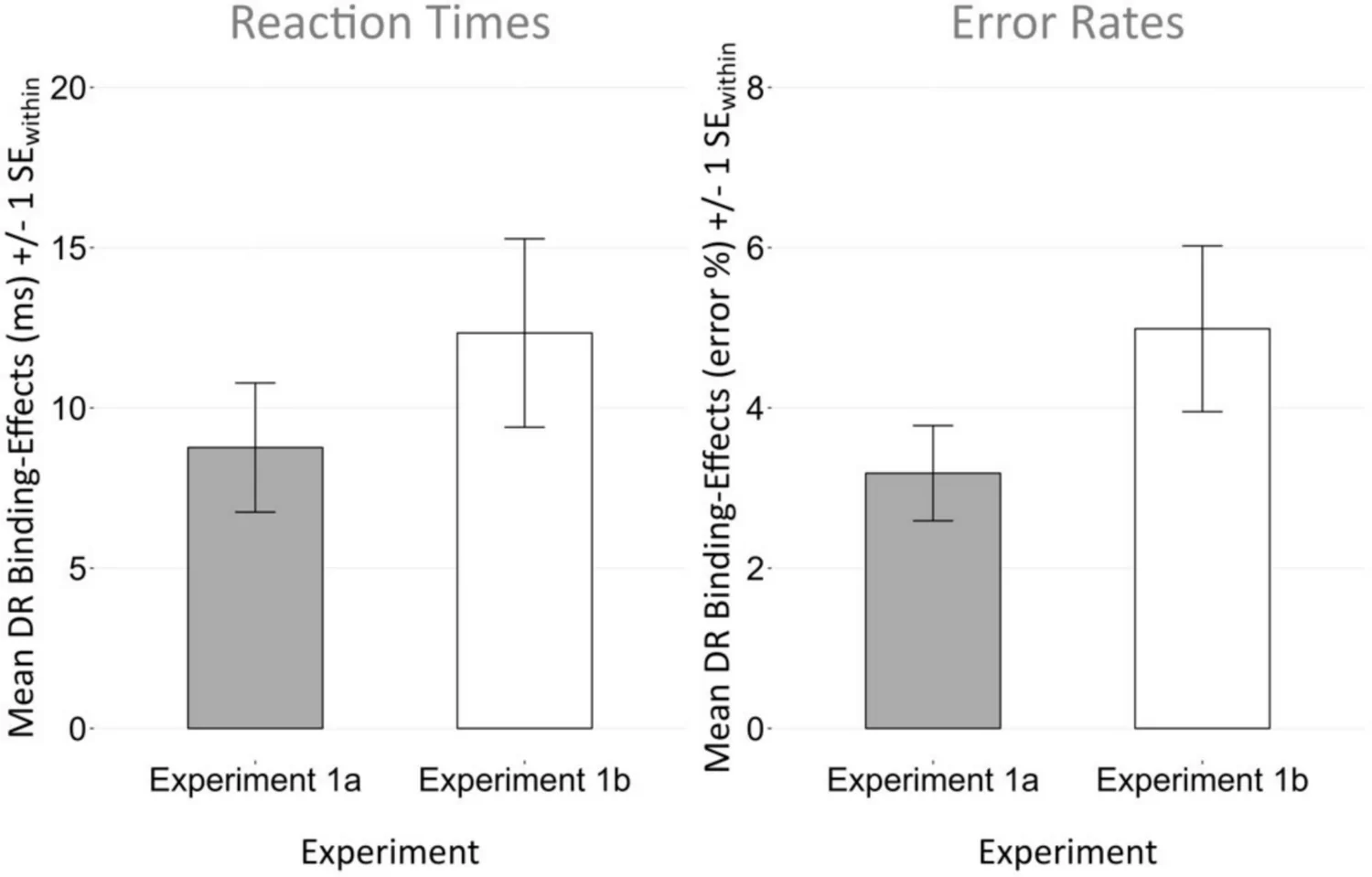
Mean binding effects in Experiments 1a and b. Distractor–response binding effects in reaction times and error rates as a function of experiment (Experiment 1a vs. Experiment 1b)

A 2 (response relation: repetition vs. change) × 2 (distractor relation: repetition vs. change) repeated-measure analysis of variance (ANOVA) on probe RTs was conducted. The main effect of response relation was significant, *F*(1,59) = 522.16, *p* < .001, ${{\eta }_{P}}^{2}$ = .48. Participants responded faster if the response repeated from prime to probe (*M* = 397 ms, *SD* = 37 ms) than if it changed (*M* = 481 ms, *SD* = 51 ms). Also, the main effect of distractor relation, *F*(1,59) = 38.20, *p* < .001, ${{\eta }_{P}}^{2}$ = .48, was significant. Participants responded faster if the distractor repeated from prime to probe (*M* = 436 ms, *SD* = 63 ms) than if it changed (*M* = 443 ms, *SD* = 59 ms). Importantly, a significant interaction between response relation and distractor relation, *F*(1, 59) = 18.93, *p* < .001, ${{\eta }_{P}}^{2}$ = .24, indicated a distractor–response binding effect (see Fig. 2).

#### Error rates

In the same analysis on error rates, the main effect of response relation was significant, *F*(1,59) = 182.83, *p* < .001, ${{\eta }_{P}}^{2}$ = .76. Participants made more errors if the response changed from prime to probe (*M* = 11.9%, *SD* = 7.3%) than if it repeated (*M* = 0.4%, *SD* = 0.8%). The main effect of distractor relation was significant as well, *F*(1,59) = 25.91, *p* < .001, ${{\eta }_{P}}^{2}$ = .31. Participants made more errors if the distractor repeated from prime to probe (*M* = 6.9%, *SD* = 8.4%) than if it changed (*M* = 5.4%, *SD* = 6.9%). Importantly, the interaction between response relation and distractor relation was significant, *F*(1, 59) = 28.76, *p* < .001, ${{\eta }_{P}}^{2}$ = .33, indicating a distractor–response binding effect.

#### Additional analysis of binding effects as a function of prime distractors

To examine whether the observed distractor–response binding effects were primarily driven by trials in which sound, but not silence, served as the prime distractor, we conducted follow-up analyses separately for sound-prime distractor and silence-prime distractor trials. When restricting the analysis to trials in which silence served as the prime distractor, distractor–response binding effects were observed both in reaction times, *t*(59) = 2.67,* p* = .010, *d*_z_ = 0.35, and in error rates, *t*(59) = 1.96,* p* = .049, *d*_z_ = 0.26. When only sound-prime distractor trials were considered, distractor–response binding effects emerged both in reaction times, *t*(59) = 2.69, *p* = .009, *d*_z_ = 0.35, and in error rates, *t*(59) = 5.62, *p* < .001, *d*_z_ = 0.72. A direct comparison of the strength of the binding effects between silence-prime distractors and sound-prime distractors revealed no difference in reaction times, *t*(59) = 0.12, *p* = .905, *d*_z_ = 0.02, and a small-sized difference in error rates,* t*(59) = 2.09, *p* = .041, *d*_z_ = 0.27, indicating slightly stronger binding effects for sound prime distractors compared to silence distractors.^1^

### Discussion

In Experiment 1a, we found evidence for distractor–response bindings that include silence as the distractor. Our findings replicated previous findings from the action control literature that event files can not only include visual but also auditory features, even if the main task is solely visual (e.g., Zmigrod et al., 2009; for a review, see Zmigrod & Hommel, 2013; see also Spence & Frings, 2020). Notably, these findings are in line with previous findings that silence is perceived as a feature (Goh et al., 2023). The current findings seem to suggest that silence was not only perceived as a feature but also bound into an event file as a feature, triggering retrieval upon repetition (see the General Discussion for alternative explanations).

However, it remains an open question whether these distractor–response binding effects including silence are comparable to regular auditory binding effects or whether they are significantly weaker. To explore this, we conducted Experiment 1b that included two different sine tones but no silence as distractors.

## Experiment 1b

### Method

#### Participants

The power analysis in Experiment 1b was identical to Experiment 1a. Sixty participants of Trier University participated in the experiment (48 women, 11 men, one not disclosed, 53 right-handed) with a median age of 21 years (range: 18–59 years). Participants were recruited via Trier University’s participant platform (Sona Systems; sona-systems.com) and performed the experiment online on the experimental platform Pavlovia (Peirce & MacAskill, 2018). All participants consented via online form before participating and received partial course credit as compensation.

#### Design

The design was the same as in Experiment 1a and included two within-subjects factors: response relation (response repetition vs. response change) and distractor relation (distractor repetition vs. distractor change).

#### Apparatus and stimulus

Apparatus and Stimuli were the same as in Experiment 1a with the difference that distractors were two sine wave tones (250 Hz or 440 Hz, created using Audacity).

#### Procedure

The procedure was identical to that in Experiment 1a with the following difference (see Fig. 1). Since both distractors were sine wave tones, a sound was presented in each prime and probe, which could either repeat or change from prime to probe.

### Results

Data processing for Experiment 1b was the same as for Experiment 1a. For the analysis of reaction times (RTs), trials with incorrect responses in prime or probe were excluded (17.3% of the trials). Additionally, we excluded 3.1% with probe RTs below 200 ms and > 1.5 interquartile ranges above the third quartile of each participant’s RT distribution (Tukey, 1977). Thus, 20.4% trials were excluded from analyses. See Table 1 for mean RTs and error rates.

#### Reaction times

A 2 (response relation: repetition vs. change) × 2 (distractor relation: repetition vs. change) ANOVA on probe RTs was conducted. The main effect of response relation was significant, *F*(1,59) = 339.79, *p* < .001, ${{\eta }_{P}}^{2}$ = .85. Participants responded faster if the response repeated from prime to probe (*M* = 397 ms, *SD* = 49 ms) than if it changed (*M* = 478 ms, *SD* = 66 ms). Also, the main effect of distractor relation, *F*(1,59) = 37.28, *p* < .001, ${{\eta }_{P}}^{2}$ = .39, was significant. Participants responded faster if the distractor repeated from prime to probe (*M* = 432 ms, *SD* = 72 ms) than if it changed (*M* = 444 ms, *SD* = 69 ms). Importantly, a significant interaction between response relation and distractor relation, *F*(1, 59) = 17.61, *p* < .001, ${{\eta }_{P}}^{2}$ = .23, indicated a distractor–response binding effect (see Fig. 2).

#### Error rates

In the same analysis on error rates, the main effect of response relation was significant, *F*(1,59) = 240.87, *p* < .001, ${{\eta }_{P}}^{2}$ = .80. Participants made more errors if the response changed from prime to probe (*M* = 15.1%, *SD* = 9.0%) than if it repeated (*M* = 1.3%, *SD* = 2.7%). The main effect of distractor relation was significant as well, *F*(1,59) = 22.50, *p* < .001, ${{\eta }_{P}}^{2}$ = .28. Participants made more errors if the distractor repeated from prime to probe (*M* = 9.4%, *SD* = 11.0%) than if it changed (*M* = 7.06%, *SD* = 7.8%). Importantly, the interaction between response relation and distractor relation was significant, *F*(1, 59) = 23.22, *p* < .001, ${{\eta }_{P}}^{2}$ = .28, indicating a distractor–response binding effect.

#### Comparison of Experiment 1a and Experiment 1b

A 2 (within: response relation: repetition vs. change) × 2 (within: distractor relation: repetition vs. change) × 2 (between: experiment: Experiment 1a vs. Experiment 1b) mixed-effects ANOVA on probe RTs yielded a significant main effect of response relation, *F*(1,118) = 830.18, *p* < .001, ${{\eta }_{P}}^{2}$ = .88. Participants responded faster if the response repeated from prime to probe (*M* = 397 ms, *SD* = 43 ms) than if it changed (*M* = 480 ms, *SD* = 59 ms). The main effect of distractor relation was significant as well, *F*(1,118) = 70.76, *p* < .001, ${{\eta }_{P}}^{2}$ = .37. Participants responded faster if the distractor repeated from prime to probe (*M* = 434 ms, *SD* = 67 ms) than if it changed (*M* = 443 ms, *SD* = 64 ms). The interaction of distractor relation and response relation was significant, *F*(1,118) = 35.06, *p* < .001, ${{\eta }_{P}}^{2}$ = .23, indicating an overall significant distractor–response binding effect. Importantly, this distractor–response binding effect was not modulated by experiment, *F*(1,118) = 1.01, *p* = .318, ${{\eta }_{P}}^{2}$ = .01, indicating no evidence for a qualitative difference in distractor–response binding effects. For the sake of completeness, the interaction of distractor relation and experiment was significant as well, *F*(1,118) = 4.28, *p* = .041, ${{\eta }_{P}}^{2}$ = .04.

In the same analysis on error rates, the main effect of response relation was significant, *F*(1,118) = 422.83, *p* < .001, ${{\eta }_{P}}^{2}$ = .78. Participants made more errors if the response changed from prime to probe (*M* = 13.5%, *SD* = 8.3%) than if it repeated (*M* = 0.9%, *SD* = 2.1%). The main effect of distractor relation was significant as well, *F*(1,118) = 44.52, *p* < .001, ${{\eta }_{P}}^{2}$ = .28. Participants made more errors if the distractor repeated from prime to probe (*M* = 8.1%, *SD* = 9.8%) than if it changed (*M* = 6.2%, *SD* = 7.4%). The main effect of experiment was significant, *F*(1,118) = 7.22, *p* = .008, ${{\eta }_{P}}^{2}$ = .06, participants made more errors in Experiment 1b (*M* = 8.2%, *SD* = 9.6%) than in Experiment 1a (*M* = 6.2%, *SD* = 7.7%). The interaction between response relation and distractor relation was significant, *F*(1, 118) = 46.91, *p* < .001, ${{\eta }_{P}}^{2}$ = .28, indicating an overall significant distractor–response binding effect. Again, this distractor–response binding effect was not modulated by experiment, *F*(1,118) = 2.28, *p* = .134, ${{\eta }_{P}}^{2}$ = .02.

A sensitivity analysis was conducted to determine the smallest detectable effect size for the between-subjects comparison of distractor–response binding effects across experiments. Assuming two independent groups with a sample size of *n* = 60 per group, a significance level of α = 0.05, and a desired power of 0.80, the analysis indicated that the experiments were powered to detect an effect size of at least *d* = 0.52.

## General discussion

The present study investigated whether silence could act as an auditory distractor in distractor–response binding. Previous studies on auditory distractor–response binding have exclusively focused on positive acoustic stimuli (e.g., sine wave tones). However, recent evidence in the literature on auditory perception suggests that silence is processed similarly to sound, in that it can be perceived and represented as an event (e.g., Skrzypulec, 2022; Sorensen, 2008). Against this background, we examined whether silence engages in binding and retrieval processes comparable to those observed for positive auditory events.

In Experiment 1a, silence (i.e., no presented sound) and a sine wave tone served as distractors. Distractor–response binding effects emerged both in reaction times and error rates. In Experiment 1b, in which distractors were two sine wave tones, distractor–response binding effects were again observed and did not significantly differ from those in Experiment 1a. Although a pure distractor–response binding effect for silence cannot be measured (since two distinct “silences” cannot be presented), the comparable magnitude of effects across experiments suggests that contrasts between two sounds and contrasts between sound and silence function similarly in binding and retrieval.

Our results on distractor–response binding effects align with recent work on the perception of silence (Goh et al., 2023), showing that silence can be represented and engage in processes typically observed for positive acoustic events. That is, silence can be integrated into and initiate retrieval from event representations, just like it was already demonstrated for positive sound distractors (Mayr & Buchner, 2006; Mayr et al., 2009, 2011a, 2011b; Moeller et al., 2012; Schöpper et al., 2020). Importantly, Goh et al. (2023) investigated silence at the level of event representations*,* demonstrating that periods of silence can be segmented and represented as auditory events that produce temporal illusions analogous to sounds. In contrast, the present work targeted silence as a stimulus feature that can be integrated into an event file, an internal representation of an action episode. Although “silence as an event representation” and “silence as a bound feature” may initially be seen as conceptually distinct, action control research assumes that features of different complexity levels (i.e., simple motor acts nested within complex goal-directed structures) can be integrated into event representations (Botvinick, 2008; Herwig et al., 2013; Lashley, 1951; Miller et al., 1960; Norman, 1981; Zacks & Swallow, 2007). For example, stimulus and response bindings in an action episode are integrated into an event representation, while at the same time successive action episodes are embedded in higher-order event representations (i.e., response–response binding; see Moeller & Frings, 2021b, 2022). From this perspective, it is conceivable that event representations of silence, as demonstrated by Goh et al. (2023), can themselves be bound into event files and therefore be subject to binding and retrieval processes.

Another important distinction concerns the operationalization of silence. In Goh et al. (2023), silence was embedded within a continuous auditory background, constituting a temporally segmented interruption. In the present study, silence was defined relative to preceding and succeeding action episodes, rather than as a disruption within an auditory stream. That is, silence was operationalized as the absence of sound in a given trial and contrasted only with primes/probes in which a sound was presented (i.e., in distractor change trials).

Contrary to the notion that silence might be represented as a *contentless* or “empty” event file (Goh et al., 2023), the present findings add to converging evidence that the cognitive system represents absence in a feature-specific rather than a vacuous manner. That is, omitted events preserve information about what is absent, not merely that something is absent. This has been demonstrated most clearly for prevention actions, where representations of prevented effects retain feature-specific information about the omitted event (Tonn et al., 2023, 2025, 2026), as well for intentionally omitted actions, which are bound and retrieved as response features although they were not executed (Nemeth et al., 2024, 2026; but see, Giesen & Rothermund, 2014; Mayr et al., 2009). Extending this to the auditory domain, our results show that silence itself can be integrated into episodic representations of action episodes, allowing it to influence subsequent behavior via the same adaptive retrieval mechanism as for positive acoustic content. Indeed, it seems that physical features are not necessary for binding effects to emerge but the mental activation of feature representations is also sufficient for nonphysical features to be represented in an event file. This activation can be achieved through visual imagery, such as imagining objects or their specific features. For instance, Cochrane and Milliken (2019) showed that imagining colors can produce event file bindings similar to those created by physical color features (see also Cochrane et al., 2023). Since vision and visual imagery are assumed to share common underlying representations (Ishai et al., 1999; O’Craven et al., 1999), these findings suggest that feature binding in both imagery and perception likely follows similar principles in action control.

In the present studies, it is important to consider whether the observed distractor–response binding effects indeed reflect the integration of silence as a *perceived* auditory feature. Alternatively, it might not have been the silence per se that was bound to and retrieved the response, but rather the contextual distinction between the presence and absence of sound. In this scenario, the trial structure, specifically, the similarity between prime and probe events, acted as the retrieval cue: A distractor repetition with silence as distractor consisted of two consecutive trials containing only a target. In contrast, a distractor change consisted of a prime trial with only a target present followed by a probe trial with both a sound and a target. Since the trial structure itself results in greater similarity between prime and probe trials for distractor repetition (both containing only the target) than for distractor change (target alone vs. target with distractor), the trial structure may have functioned as a strong retrieval cue (Benini et al., 2022; Koch et al., 2018; Laub & Frings, 2020a; Qiu et al., 2023). In other words, the contextual distinction between “sound present” and “sound absent” may have operated as a feature that was bound to the response and later retrieved it, without requiring silence to be encoded as a physical auditory stimulus in the same sense as a presented tone. Thus, the present data do not warrant the strong claim that silence is bound as a physical auditory stimulus in the same way as positive acoustic content. Rather, they are fully compatible with an interpretation in terms of a contextual contrast between the presence and absence of sound.

Importantly, however, this contextual contrast was induced by the manipulation of silence. Thus, either the perceived silence itself or the experimental context (i.e., the presence vs. absence of sound) acted as a retrieval cue; in both cases, the silence manipulation served as the critical trigger for retrieval. This means that irrespective of whether silence was construed as a perceptual auditory event or as a contextual feature, the presence–absence distinction on the auditory dimension was encoded and operated as a feature, modulating action control through distractor–response binding effects as previously only observed for positive acoustic content.

This interpretation also constrains concerns about overly broad or unconstrained binding of “absent” features. Within binding and retrieval accounts, features are not limited to physical stimulus properties but comprise any discriminable distinction that is represented by the cognitive system in a given task context (Münster & Frings, 2025). In the present experiments, the presence versus absence of sound constituted a salient and task-structured dimension that systematically differentiated prime and probe events. Accordingly, we do not assume that absence is bound indiscriminately across modalities or situations (e.g., absent smell or taste). Rather, our findings suggest that when an experimental context establishes a relevant contrast on a specific dimension, such as auditory presence versus absence, this distinction can engage in event-file binding and retrieval.

Finally, several empirical observations argue against the interpretation that the observed effects in Experiment 1a were driven exclusively by sound-prime trials. If silence-prime episodes did not contribute to binding at all, distractor–response binding effects in Experiment 1a should have been smaller relative to Experiment 1b, because only a subset of prime trials would be subject to binding in the former. The absence of any reliable difference in the strength of binding effects between the two experiments is inconsistent with this prediction. A related possibility is that the relative rarity and thus the higher salience of the sound in Experiment 1a might have selectively boosted the binding effect for prime sound trials, thereby producing an overall comparable effect to the two sounds in Experiment 1b. Although salience has been shown to modulate the binding of task-relevant features (e.g., Schmalbrock et al., 2021), more recent evidence suggests that distractor–response binding effects are not influenced by salience and that salience primarily affects binding when the respective feature is task-relevant (Laub & Frings, 2024). Against this background, a salience-based account is unlikely to explain the comparable binding effects observed across the two experiments. Crucially, in addition, more fine-grained analyses within Experiment 1a revealed binding effects even when restricting the data set to silence-prime trials, indicating that the overall binding effect was not driven solely by sound-prime episodes.

In summary, whether construed as a perceptual auditory event or represented as a differentiable experimental context as the presence versus absence of sound, silence in the present paradigm was represented, bound, and retrieved as a feature and affected behavior in exactly the same way as positive acoustic content. Thus, our findings support the assumption of differentiated event representations of absence (Goh et al., 2022) and in turn that silence can indeed affect cognitive processes in action control.

## Conclusion

In conclusion, by using silence (i.e., the absence of sound) and sound as a distractor, we investigated whether silence features can be bound to responses and subsequently initiate their retrieval upon being repeated. Previous research suggested that if silence is actually perceived it should be treated just as any other (here auditory) feature by our cognitive system. Notably, the repetition of silence and sound as distractors elicited distractor–response binding effects that were in comparable strength to those observed when only concrete auditory stimuli were used. This suggests that silence was cognitively processed as an abstract feature capable of being bound to responses and later retrieving them. The findings show for the first time that moments of absence (i.e., perceived silence) influence action control in exactly the same way as positive acoustic content.

## Data Availability

The data of the experiments is available at PsychArchives (10.23668/psycharchives.15264). None of the experiments was preregistered.

## Footnotes

1 Note that this difference in distractor–response binding effects for prime distractors in error rates is consistent with the assumption of graded differences in feature strength, but not with an account in which silence is not bound and retrieved as a feature.

## Declarations

Data of the experiments are available at PsychArchives (https://doi.org/10.23668/psycharchives.15264), and none of the experiments was preregistered.
